# Efficacy and safety of citrate-based anticoagulation compared to heparin in patients with acute kidney injury requiring continuous renal replacement therapy: a randomized controlled trial

**DOI:** 10.1186/s13054-015-0822-z

**Published:** 2015-03-18

**Authors:** Fabien Stucker, Belen Ponte, James Tataw, Pierre-Yves Martin, Hannah Wozniak, Jérome Pugin, Patrick Saudan

**Affiliations:** Nephrology Unit, Geneva University Hospitals, Rue Gabrielle-Perret Gentil 4, Genève, 1205 Switzerland; Intensive Care Unit, Geneva University Hospitals, Rue Gabrielle-Perret Gentil 4, Genève, Switzerland

## Abstract

**Introduction:**

A systemic anticoagulation is often required to prevent circuit and filter clotting in ICU patients undergoing continuous renal replacement therapy (CRRT). A regional citrate-based anticoagulation (RCA) does not induce a systemic anticoagulation and prolongs the filter lifespan, but metabolic side-effects have been associated with this therapy. We conducted a randomized controlled trial with patients requiring CRRT to determine whether RCA using a balanced predilution replacement fluid is more effective than heparin in terms of renal replacement delivered dose and safety profile.

**Methods:**

One hundred and three patients with AKI requiring CRRT were included. The patients were randomized to either CRRT with RCA or heparin anticoagulation. Primary endpoints were effective daily delivered RRT dose during the first 3 days of CRRT and filter lifespan. Secondary endpoints were 28-day and 90-day survival and severe metabolic complications and bleeding disorders.

**Results:**

Median CRRT duration was 3.0 (2–6) days. Effective delivered daily RRT doses were 29 ± 3 and 27 ± 5 mL/kg/hr in the RCA and heparin groups, respectively (p = 0.005). Filter lifespans were 49 ± 29 versus 28 ± 23 hrs in the RCA and heparin groups (p = 0.004). Survival rates at 28 and 90 days were 80-74% in the RCA and 74-73% in the heparin group. Electrolytes and acid–base disturbances were uncommon and transient in patients treated with RCA.

**Conclusions:**

These results show that RCA is superior to heparin-based anticoagulation in terms of delivered RRT dose and filter life span and is a safe and feasible method. This does not translate into an improvement in short term survival.

**Trial registration:**

ClinicalTrials.gov NCT01269112. Registered 3rd January 2011.

## Introduction

Acute kidney injury (AKI) is a common complication in the ICU setting, occurring in nearly 5 to 7% of the patients and burdened by a high mortality rate [1]. Renal replacement therapy (RRT) is needed in 70% of ICU patients with AKI and continuous renal replacement therapy (CRRT) is implemented in 80% of the cases [1]. Systemic anticoagulation is often required to prevent clotting of the filter and extracorporeal circulation. Until recently unfractionated heparin was the standard and the most-used anticoagulation therapy in the ICU setting [2]. However, ICU patients are at higher risk of bleeding for many reasons (surgical procedures, trauma, liver dysfunction, thrombocytopenia), and this risk is increased when systemic anticoagulation is used.

By chelating calcium, citrate inhibits the clotting cascade and thrombin generation, and can therefore be used to specifically anticoagulate the extracorporeal circulation and filter during CRRT. The use of postfilter calcium supplementation is necessary to restore normal systemic calcium levels and full systemic coagulation [3,4]. Citrate can induce severe hypocalcemia, as well as other metabolic disorders such as metabolic alkalosis or acidosis, especially acidosis in patients with severe liver impairment as citrate is mainly metabolized into bicarbonate by the liver. To avoid these serious side-effects, protocols of regional citrate administration have been developed along with the use of postfilter and systemic ionized calcium measurements. This enables modulation of citrate flow rate within the extracorporeal circuit, as well as calcium supplementation, in order to maintain anticoagulation in the circuit and normal systemic calcium levels.

Some human studies have demonstrated that regional citrate-based anticoagulation (RCA) may extend the filter lifespan and therefore minimize filter clotting, circuit downtime and blood losses [5-9]. A decrease in mortality has also been observed in the largest RCT published to date, although this was not confirmed in a subsequent trial [10,11].

According to the new Kidney disease improving global outcomes (KDIGO) guidelines, RCA should be now the first choice for CRRT anticoagulation [12]. Due to time-consuming procedures and a more complex protocol, its implementation within the ICU setting can however, be more difficult than use of standard heparin. Citrate can be administered prefilter, either as a separate solution, or contained within a balanced predilution replacement solution.

As this modality may be more caregiver-friendly in terms of implementation, we conducted a randomized controlled trial in ICU patients requiring CRRT to determine whether a balanced predilution replacement fluid with citrate was more effective than heparin in terms of delivered renal replacement dose, filter lifespan, safety profile and patient survival.

## Methods

### Study design and outcomes

The study was a monocentric prospective open-label randomized controlled trial at the ICU of the University Hospitals of Geneva (Switzerland). The study was approved by the local ethical committee from the Geneva University Hospitals and registered at ClinicalTrials.gov (NCT01269112). A consent form was obtained from all enrolled patients, their next-of-kin or a senior ICU physician who was neither in charge of the patient nor involved in the study. Consent was sought and confirmed whenever patients regained decision-making capacity.

### Setting and patients

The Geneva University Hospitals ICU is a 36-bed unit taking care of medical, surgical, trauma, and transplant patients (n = 2,600 admissions/yr). CRRT indications and implementations are under the supervision of intensive care physicians and nephrologists with the involvement of the nephrology nurses for the CRRT set up.

### Inclusion criteria

ICU patients were eligible if they were ≥18 years of age and had an AKI requiring CRRT according to the kidney-failure criteria of the RIFLE definition [13].

### Exclusion criteria

Patients were excluded if they had active hemorrhagic disorders or severe thrombocytopenia (<50 × 10^9^/L), a history of heparin-induced thrombocytopenia, severe liver failure defined as a factor V <20%, or were on the waiting list for liver transplantation.

### Treatment assignment

Subjects enrolled into the trial were randomly allocated to either heparin or citrate anticoagulation. A randomization list was generated by computer in random blocks of five patients, and blinded for the investigators. Sealed, opaque and sequentially numbered envelopes with the respective allocation cards were prepared by the Unit of Quality Care. The on-call nurse from the Nephrology Unit opened the next available envelope each time a patient was enrolled in the study. Blinding was impossible to perform for obvious logistic reasons. A unique identification code was assigned to the subject at inclusion. Data were collected and analyzed using this anonymous number.

### Intervention

CRRT was performed in both arms by pump-driven devices (Prismaflex-Gambro Lundia, Lund, Sweden) with fluid balance systems and a biocompatible high-flux membrane measuring 1.5 m^2^ (ST-150; Gambro). Continuous veno-venous hemodiafiltration (CVVHDF) was started at a dose of 30 ml/kg/h, of which 10 ml/kg/h were dialysate flow. Two thirds of the replacement fluids were administered in the predilution mode and one third in the postdilution mode. Our dose protocol was tailored to match a 25 ml/kg/h dialysis dose obtained in the postdilution mode in order to compensate for the lower efficacy of our 2/3 predilution protocol. As our predilution reinjection flow rate adds 16% more fluid to our blood flow rate, we estimated that 30 ml/kg/h would be equivalent to the dialysis dose implemented in the renal trial [14]. The ultrafiltration rate was adapted by the ICU team according to clinical criteria, with a recommendation not to exceed 200 ml/kg/h. In the absence of clotting, the filter was changed after 72 h following manufacturer’s recommendations. A double-lumen catheter was inserted through a central vein. A triple-lumen catheter was used when no other central venous line was available for calcium infusion. Blood flow was maintained between 100 and 200 ml/min. All solutions except Prismocitrate contained bicarbonate.

### Citrate

CVVHDF was performed using Prismocitrate 18/0 solution (Trisodium citrate 18 mmol/L, Na^+^ 136 mmol/L, Cl^−^ 86 mmol/L) in the predilution mode, Prismocal B22 (Mg^2+^ 0.75 mmol/L, Na^+^140 mmol/L,K^+^ 4 mmol/L, Cl^−^120.5 mmol/L, lactate 3 mmol/L HCO_3_- 22 mmol/L) in the dialysate mode, and Prismasol (Ca^2+^ 1.75 mmol/L, Mg^2+^ 0.5 mmol/L, Na^+^ 140 mmol/L, Cl^−^113.5 mmol/L, lactate 3 mmol/L HCO_3_- 32 mmol/L, K^+^ 4 mmol/L, glucose 6.1 mmol/L) in the postdilution mode. All the solutions were made and delivered by Gambro. The protocol was designed to adjust the citrate solution flow rate to the patients’ blood flow rate to target a blood citrate concentration of 3 mmol/L. Blood flow was therefore maintained between 100 and 200 ml/min according to the patient’s body weight in order to achieve this target. In the case of early signs of clotting, citrate dose was further adapted to aim for a postfilter ionized calcium of 0.25 to 0.3 mmol/L. Postfilter ionized calcium was measured 15 minutes after any change in blood, reinjection, dialysate, or calcium flow rates.

A protocol was followed by the intensive care nurses (ionized systemic calcium and bicarbonate were measured by arterial blood gases every 3 h during the first 24 h, then every 5 h) to adapt the dialysate flow to maintain the blood pH within normal range, and to adapt the postfilter calcium administration to prevent systemic hypocalcemia.

### Heparin

CVVHDF was performed using unfractionated heparin as anticoagulant and Prismasol as reinjection and dialysate fluids. The dose of heparin was prescribed by the intensive care physician depending on the patient’s medical condition. A minimal dose of 500 UI/h was required to assure circuit patency. The treatment was continued until recovery of renal function, which was defined as a urine output of ≥1 ml/kg/h or stable plasma creatinine values 24 h after CRRT discontinuation, or the start of intermittent hemodialysis or death. Treatment was stopped in the case of any adverse event possibly related to the type of anticoagulation. The event was signaled and treatment resumed or switched to the other mode of anticoagulation, according to the judgment of the ICU physician in charge of the patients.

### Study endpoints

We assessed the following parameters during RRT days: filter lifespan (duration of use until non elective circuit disconnection due to filter clotting or effective transmembranous pressure >300 mmHg); daily delivered RRT dose (ml/kg/h), corresponding to the h/day when the prescribed RRT dose was delivered (including filter downtime), a daily 30 ml/kg/h RRT dose being a delivered RRT dose during 24 h without downtime; daily effective delivered RRT dose (ml/kg/h) corresponding to the h/day when prescribed RRT dose was delivered (excluding elective filter downtime, such as treatment interruptions for radiological or surgical procedures); bleeding episodes requiring transfusions; episodes of heparin-induced thrombocytopenia (HIT); and metabolic disorders (defined as metabolic alkalosis with pH >7.55, metabolic acidosis with pH <7.25, clinically relevant hypocalcemia with ionized calcium <1 mmol/L, citrate accumulation defined as a Ca tot/Ca ion ≥2.5).

We assessed the following parameters during follow-up: vital status at 28 and 90 days (survival, hospitalization and requirement of maintenance RRT); primary outcomes of mean daily delivered RRT dose during the first 3 days of CRRT (ml/kg/h) and filter lifespan; secondary outcomes of patient survival at 28 and 90 days, length of ICU stay, bleeding episodes, occurrence of HIT and severe metabolic disorders. Survival status was assessed according to the Hospital database linked with the Geneva State Registry office.

### Statistics

Statistics were performed using SPSS18 software package (SPSS Inc., Chicago IL, USA). All categorical variables are presented as number and percentage and all continuous variables with a normal distribution as mean ± SD. When not normally distributed, variables are expressed as median and IQR. Parametric and non-parametric tests were used to compare baseline characteristics of study groups. For the primary outcome (filter lifespan), survival analysis was assessed with Kaplan-Meier curves and groups were compared by log rank test. Analyses were conducted on an intention-to-treat basis. A two-side *P*-value <0.05 was considered significant.

### Sample size

We hypothesized that the effective daily CRRT dose would be 95 ± 10% of the prescribed dose with citrate-based replacement fluid and 75 ± 20% with heparin. We calculated a sample size of 49 patients per arm to obtain 80% power with a two-sided α level <0.05 and to detect a 20% change in the prescribed dose effectively delivered (primary outcome). The trial was stopped after randomization of 103 patients.

## Results

**Figure 1:**
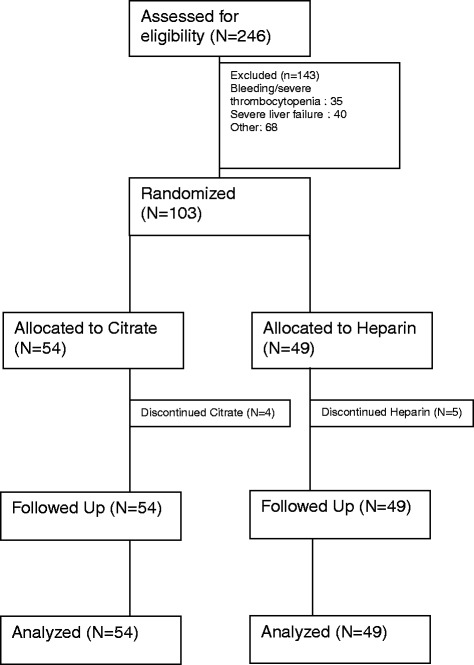
**Flow chart of the trial.**

From October 2011 to July 2013, we screened 246 patients with AKI requiring CRRT within the Medical and Surgical ICU of the Geneva University Hospitals. We enrolled 103 of them in the study, and they received the allocated treatment. Reasons for non enrollment for the remaining 143 patients are mentioned in the study flow chart (Figure 1).

Demographic and clinical characteristics at baseline in both arms are shown in Table 1. Baseline characteristics were well-matched across groups.

**Table 1:** **Baseline characteristics of the participants according to randomization**

**Variables**	**Citrate**	**Heparin**	***P***
	**(n = 54)**	**(n = 49)**	
**Age**	60 (14)	65 (16)	0.07
**Male gender**	32 (59)	32 (64)	0.55
**Weight, kg**	82 16)	80 (18)	0.67
**Diabetes**	19 (36)	16 (33)	0.73
**Chronic kidney disease**	22 (42)	17 (35)	0.48
**Coronary artery disease**	9 (17)	13 (27)	0.24
**Cerebrovascular disease**	4 (8)	4 (8)	0.91
**Cardiac heart failure**	13 (25)	15 (30)	0.49
**Chronic liver disease**	6 (11)	6 (12)	0.89
**Cancer**	6 (11)	10 (20)	0.21
**Diagnosis of renal failure**			
(medical/trauma/surgical)	44 (81)/3 (5)/7 (13)	35 (71)/1 (2)/13 (27)	0.17
**Laboratory data**			
Serum creatinine, μmol/L	471 (319)	455 (296)	0.89
BUN, mmol/L	27 (17)	26 (19)	0.80
Hemoglobin, g/L	104 (22)	107 (22)	0.52
Platelets, 10^9^/L	217 (160)	181 (115)	0.20
INR	1.2 (0.3)	1.3 (0.4)	0.70
PTT, sec	46 (23)	53 (39)	0.24
pH	7.27 (0.16)	7.28 (0.14)	0.61
Bicarbonate, mmol/L	18.3 (5.4)	16.4 (5.9)	0.37
Total calcium (mmol/L)	2.31 (0.24)	2.28 (0.23)	0.51
Ionized calcium (mmol/L)	1.13 (0.12)	1.12 (0.11)	0.54
**Severity score data**			
Sepsis	32 (60)	31 (63)	0.76
Oliguria	34 (69)	35 (71)	0.83
Inotropic support	31 (60)	30 (61)	0.87
Mechanical ventilation	32 (62)	28 (57)	0.65
APACHE II score	28 (9)	29 (9)	0.58
SAPS score	63 (18)	65 (18)	0.61

Data are expressed as mean (SD). Categorical data are expressed as number (%). BUN, blood urea nitrogen; INR, internal normalized ratio; PTT, partial thromboplastin time; APACHE, acute physiology and chronic health evaluation; SAPS, simplified acute physiology score.

### Primary outcome

**Figure 2:**
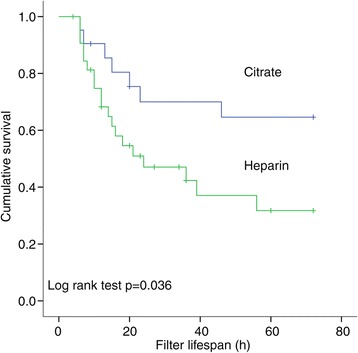
**Kaplan-Meier analysis of the filter lifespan.**

Mean daily delivered RRT dose for patients during RRT were 29 ± 3 ml/kg/h in the RCA group and 27 ± 5 ml/kg/h in the heparin group (*P* = 0.005). In the 52 patients who did not need to have RRT stopped for elective reasons during the first 3 days of RRT, the mean daily delivered RRT dose for was 29 ± 5 ml/kg/h in the RCA group and 25 ± 4 ml/kg/h in the heparin group (*P* = 0.007), and the mean filter lifespan significantly increased in the RCA group compared to the heparin one (49 ± 29 versus 28 ± 23 h, vs = 0.004) (Figure 2).

### Secondary outcomes

#### Safety issues

Four patients in the citrate group were switched to heparin during the study: one on account of worsening liver failure, one on account of a technical problem with calcium infusion, and two for clinically relevant hypocalcemia (one with concomitant intractable severe metabolic acidosis due to septic shock and one whose treatment was changed by the team for no clear reasons). In the six patients with RCA who had severe hypocalcemia, mean total calcium was 1.78 (0.10) mmol/L, ionized calcium was 0.94 (0.27) mmol/L and Ca tot/calcium ion ratio was 2.17 (1.20). Citrate accumulation, identified as a Ca ratio (total calcium/ionized calcium >2.5), was only observed in one patient and was transient, as ionized hypocalcemia responded well to increased calcium supplementation. Five patients were switched from heparin to citrate: two patients with major bleeding, and three because of recurrent filter clotting. Metabolic disorders and episodes of bleeding are listed in Table 2.

**Table 2:** **Intervention data**

**Variables**	**Citrate**	**Heparin**	***p***
	**(n = 54)**	**(n = 49)**	
**Delivered RRT dose, ml/kg/h**	29 (3)	27 (5)	**0.005**
Effective delivered RRT dose*, ml/kg/h	28 (5)	26 (4)	0.15
**Filter lifespan, h**	49 (29)	28 (23)	**0.004**
**Mean heparin, IU/ml dose**	6,757 (5,455)	10,567 (7,760)	0.005
**Laboratory follow-up data**			
Total calcium, mmol/L, day 1	2.34 (0.20)	2.31 (0.19)	0.56
Ionized calcium, mmol/L, day 1	1.05 (0.10)	1. 12 (0.09)	0.04
pH, day 1	7.32 (0.10)	7.31 (0.11)	0.62
Bicarbonate, mmol/L, day 1	18 (4.6)	19 (7.2)	0.96
Na, mmol/L, day 1	136 (15)	138 (7)	0.42
Chloride, mmol/L, day 1	104 (15)	108 (7)	0.15
Potassium, mmol/L, day 1	6 (14)	5.3 (5.6)	0.61
Lactate, mmol/L, day 1	1.3 (0.9 to 2.9)	1.3 (0.8 to 1.8)	0.67
Total calcium, mmol/L, day 3	2.52 (0.19)	2.41 (0.22)	0.02
Ionized calcium, mmol/L, day 3	1.14 (0.10)	1.20 (0.11)	0.01
pH, day 3	7.40 (0,06)	7.41 (0.06)	0. 39
Bicarbonate, mmol/L, day 3	23.71 (1.81)	25.17 (4.31)	0.43
Na, mmol/L, day 3	138 (3.37)	138 (4)	0.71
Chloride, mmol/L, day 3	104 (3.4)	107 (4)	0.00
Potassium, mmol/L, day 3	4 (0.52)	4.3 (0.6)	0.03
Lactate, mmol/L, day 3	1.4 (0.9 to 2.2)	1.1 (0.9 to 1.4)	0.50
**Side effects**	32	27	0.17
Bleeding	0	4 (8)	
HIT	1 (2)	2 (4)	
Filter clotting	3 (6)	18 (37)	
Metabolic disorders:	14	3	
Metabolic alcalosis	3	0	
Respiratory alkalosis	0	1	
Metabolic acidosis	3	1	
Severe hypocalcemia	6	1	
Ca total/calcium ion ratio >2.5	1	0	
**CRRT, days**	3 (2 to 6)	3 (2 to 5)	0.30
**ICU, days**	7 (4 to 15)	7 (4 to 12)	0.79
**Hospital, days**	22 (6 to 35)	16 (9 to 30)	0.45
**Survival at 28 days**	43 (80)	36 (74)	0.46
**Survival at 90 days**	40 (74)	35 (73)	0.90

Data are expressed as mean (SD) or median (IQR) according to the distribution, and categorical data are expressed as number (%). *Including elective filter downtime. RRT, renal replacement therapy; CRRT, continuous renal replacement therapy.

### Mortality

In the intention-to-treat analysis, 28- to 90-day mortality rates were 20 to 26% and 26 to 27% in the citrate and heparin groups, respectively (*P* = 0.37). In the per protocol analysis, 90-day mortality rates were 27% and 30% in the citrate and heparin groups, respectively (*P* = 0.33). The 90-day mortality rate in the 246 critically ill patients treated by CRRT during the study period was 38%.

### Length of CRRT and stay

RRT duration was slightly longer for patients in the heparin group, although the difference did not reach statistical significance (Table 2). Median duration of ICU stay was similar in both groups. Median duration of hospitalization was slightly longer in the citrate group (Table 1).

### RRT long-term dependence

Nine patients remain RRT-dependent, five in the heparin group, and four in the citrate group at 90-day follow up.

### Subgroup of patients with liver failure

Twelve patients with liver failure, defined as previously known to have cirrhotic disease or acute elevation of amino transferases associated with prolonged clotting time and encephalopathy, in whom factor V was still >20% at screening, have been enrolled in the study. Among these patients, six were randomized into the citrate group, of whom four survived >90 days. RCA was well-tolerated and implemented without any subsequent metabolic disorders in these patients.

## Discussion

Since the initial publication by Mehta *et al*., RCA has slowly gained support among nephrologists and intensivists treating ICU patients with AKI requiring CRRT [15]. However, its widespread use is hampered by fears of severe metabolic side-effects, such as citrate accumulation leading to hypocalcemia and acid-base disorders [7,16]. Our results show that citrate-based regional anticoagulation is safe, and that metabolic complications are rare when a standardized protocol is used to adapt dialysate flow and calcium substitution in order to maintain blood pH and ionized calcium levels within the normal range.

We used a commercially available balanced predilution replacement solution, with an administered volume coupled to blood flow in order to minimize caregiver-induced manipulation errors. Filter lifespan and thus, effective daily RRT dose, were significantly increased with this RCA protocol. RCA dramatically decreases the filter clotting, which is a frequent complication of CRRT, especially in patients with acute critical illness such as sepsis, where thrombogenicity is increased [17]. An increased filter lifespan means less treatment interruption and more effective dialysis time. One of the most frequent problems encountered in these patients treated by CRRT is indeed circuit downtime, implying that delivered RRT dose is often lower than prescribed [18]. We found that filter clotting occurred only in 6% of the patients within the RCA group versus 37% of the patients within the heparin group. This favorable effect was shown in many, but not all, prior publications which are encompassed in two recent meta-analyses, leading to the sound conclusion that citrate may be more effective that heparin in terms of filter lifespan [5,19-22].

A few studies have evaluated the safety and efficacy of custom-made citrate solutions. In a recent multicenter randomized study, a custom-made calcium-free trisodium citrate replacement fluid (13.3 mmol citrate/L) was compared with bicarbonate- and lactate-based replacement solutions in ICU patients [8]. No difference was found for 28- and 90-day mortality when using citrate as compared to bicarbonate- and lactate-based replacement solutions. Six percent of the patients randomized to RCA had citrate accumulation, which led to RCA interruption, as compared to one third of heparin interruption within the other group. Citrate was found to be superior in terms of safety and cost-effectiveness. The lower rate of citrate accumulation in our patients is probably due to the fact that our patients represented a different population, as patients with severe liver failure were excluded.

In an uncontrolled retrospective study with a citrate-based commercially available solution, 16 cases of citrate accumulation and 4 cases of CVVH termination due to citrate accumulation were reported [23]. This unexpectedly high rate of complication may have been related to the very high CVVH dose (45 ml/kg/h) that was used in this study. Moreover, the necessity of an exogenous infusion of sodium bicarbonate may have increased the complexity of the procedure.

Another prospective observational uncontrolled trial has recently been conducted to assess the safety of a custom-made, not yet commercially available, citrate solution [24] in patients prone to bleeding. CVVH with RCA was found to be safe in these patients, and citrate had to be withdrawn in only 11% of the patients, especially in patients with higher transaminases. This rate is higher than in our study, probably related to the number of patients with moderate to severe liver disease in this study and to the lower citrate flow that we used.

We had no bleeding within the RCA group but slightly more metabolic side effects. The clinical impact was minor as only two patients had to be switched to heparin on account of severe hypocalcemia. In comparison, five patients in the heparin group had to be changed to RCA on account of clinically significant bleeding or recurrent filter clotting. In terms of safety issues, RCA seems therefore to have a favorable profile.

In the subgroup of 12 patients with liver failure but with factor V >20%, RCA treatment was tolerated as well as heparin. Due to the small number of patients, it is difficult to draw any conclusion about the safety of RCA in this subset of patients. Moreover, we excluded the patients with the worst liver function (defined as a factor V <20%). Results from a prospective observational study investigating citrate accumulation in patients with decompensated liver cirrhosis or acute liver failure were recently published. Twenty-eight patients received citrate-based CCVHDF and even though these patients had a higher total calcium/ionized calcium ratio, which reflects citrate accumulation, major disturbances of acid-base or electrolyte status were not found [25].

Although this was not our primary objective, we were unable to show a significant reduction in mortality with RCA use, as observed in the largest published RCA trial (7). This positive effect of citrate anticoagulation on survival was noted especially in patients with sepsis and in patients with higher severity scores. In this trial, the 90-day mortality was 63% in the nadroparin group versus 48% in the RCA group. This survival advantage however, was not observed in another recently published RCT, where 174 patients requiring RRT were randomized to RCA versus standard heparin [8]. Different explanations could account for the discrepancies on mortality between our results and those of the Dutch trial [7]. First, the overall mortality (20 and 23.9%) is remarkably low in our trial and clearly lower than what has been described so far in similar populations. Lower mortality seems to be common in recently published RCTs in the ICU setting, most probably reflecting improvement in the standard of care. Second, our sample size does not allow us to detect a significant change in mortality. Mean age and percentage of surgical patients and those on mechanical ventilation were also higher in the Dutch trial than in our trial, which could also explain the highest mortality rate. Finally, low-molecular-weight heparin was used in that trial compared with the unfractionated heparin in our study.

Our study has several limitations. First, it is relatively small, therefore underpowered to detect beneficial effects other than filter lifespan and more efficient delivery of the RRT dose. It is monocentric and cannot be generalized to other ICUs, as our patient management involved two medical teams of nephrologists and intensivists working closely together. Second, a direct measurement of the dialysis dose with urea clearance could not be measured for practical reasons, and we used the delivered RRT dose calculated on treatment duration as a proxy for dialysis efficiency.

## Conclusion

Notwithstanding, our results show that metabolic complications of RCA can be avoided with the help of a strict protocol and the use of a commercially available predilution citrate fluid, with a safety profile that looks promising. We also confirm that hemorrhagic complications can be avoided with RCA. Although RCA use did not translate into an improvement in 90-day survival in our trial (which was underpowered for this endpoint), there is a clear advantage of RCA over heparin-based anticoagulation in terms of filter lifespan and effective daily delivered RRT dose.

## Key messages

- In ICU patients with AKI treated with continuous RRT, regional citrate anticoagulation is superior to heparin in terms of filter life span and delivered RRT dose
- Metabolic complications of RCA can be avoided with the help of a strict protocol

## Abbreviations

AKI: acute kidney injury
CRRT: continuous renal replacement therapy
CVVHDF: continuous veno-venous hemodiafiltration
HIT: heparin-induced thrombocytopenia
KDIGO: Kidney disease improving global outcomes
RCA: regional citrate-based anticoagulation
RRT: renal replacement therapy
